# Analyzing the morphology and avian β-defensins genes (AvβD) expression in the small intestine of Cobb500 broiler chicks fed with sodium butyrate

**DOI:** 10.1186/s12917-024-04253-y

**Published:** 2024-09-28

**Authors:** Mohamed A.M. Alsafy, Islam A. Abdellatif, Samir A. A. El-Gendy, Mohamed M.A. Abumandour, Ahmed Noreldin, Naglaa F. Bassuoni

**Affiliations:** 1 Department of Anatomy and Embryology, Faculty of Veterinary Medicine, Alexandria University, Abis 10th, P.O. 21944, Alexandria, Egypt; 2 Histology and Cytology Department, Faculty of Veterinary Medicine, Damanhour University, Damanhour, 22511 Egypt

**Keywords:** Chicken, Small intestine, Sodium butyrate, SEM, Real-time PCR, AvBD

## Abstract

**Background:**

Sodium butyrate is a potential antibiotic growth promoter and has had advantageous effects on the poultry industry.

**Methods:**

Evaluating the effect of sodium butyrate on the intestinal villi and the humoral part of innate immunity of the male Cobb 500 broiler using scanning electron microscopy and quantitative real-time PCR analysis, the control group and treated group of Cobb 500 with SB supplemented received water containing 0.98 mg sodium butyrate.

**Results:**

The administration of sodium butyrate changed the villi characters, as the shape changed from tongue to long tongue. They were mainly parallel to each other and long finger-like at the duodenum. The tips of the villi in the control group appeared thin-slight curved with a prominent center in the duodenum, thin rectangular in the jejunum, and ileum in the control group. In contrast, in the treatment group, they changed to thick rectangular in the duodenum and ileum zigzag shape in the jejunum. The epithelium lining of the duodenal villi showed a dome shape, the jejunal villi showed a polygonal shape, and the ileal villi appeared scales-like. The epithelium lining showed irregular microfolds and many different-sized pores, and the treatment group showed islands of long microvilli in the duodenum and solitary long microvilli in the ileum. Real-time PCR of AvBD 1, 2, 10, and 12 significantly (*P* < 0.01). The better expression of AvBD 1, 2, and 12 was determined in the duodenum, while AvBD 10 was in the jejunum.

**Conclusion:**

Sodium butyrate enhanced the chicks’ growth and small intestine parameters, modified the morphology of the intestinal villi, and improved the humoral part of innate immunity.

## Introduction

The intestinal mucosa is exceptionally convoluted and specialized for the maximal absorption of nutritional additives [1]. The epithelium folds into villi, and epithelial cells have an apical component, including dense matting of microvilli forming a broom border, which increases the small intestinal floor region for absorption by approximately six hundredfold, resulting in improved nutrient absorption [2, 3]. The nutritional value of diets may produce microscopic alterations in the intestinal mucosa [2, 3]. Numerous studies have investigated the ultrastructure of the chick intestine [4–6].

The sodium butyrate (SB), is regarded as a potential growth promoters (AGP) alternative due to its advantageous effects on the poultry industry [7, 8]. Because butyric acid has a disagreeable odour and potentially unstable volatility of butyric acid, sodium butyrate has generally been used in broiler production [9]. In the gastrointestinal system of chickens, sodium butyrate is easily converted into an effective component. The development of the intestinal mucosa and morphological structures are enhanced by sodium butyrate supplementation, and the growth of the symbiotic intestinal microbiota is thought to be moderated. As a result, dietary SB may be beneficial for the physiological function and health of the intestines [10].

Defenses are antimicrobial peptides that can cause an innate immune reaction and have been divided into three groups, specifically α−, β−, and Θ-defenses. Avian antimicrobial peptides categorized as β-defensins were formerly known as gallinacins. However, it has now been agreed to apply their gene avian β-defensin [11]. Thirteen avian β-defensins genes (AvβD) have been recognized. The expression of the three sorts of AvβD had been proven inside the oviduct. If the synthesized avian β-defensins play roles inside the host immunity to dispose of microorganisms, their expressions are predicted to be more advantageous in reaction to bacterial additives. Host protection peptides (HDPs) represent a massive organization of herbal broad-spectrum antimicrobials, and a crucial first line of immunity is simply all kinds of life [12].

In this study, sodium butyrate is used to assess the impact of 0.98 mg on β-defensin genes (AvβD) and innate immunity. That could help with using SB in our country’s routine feeding program. So, the current study used scanning electron microscopy and quantitative real-time PCR to determine the effect of sodium butyrate on the intestinal villi microanatomy and the humoral part of the Cobb 500’s innate immunity in a healthy broiler.

## Materials and methods

### Animals and experimental design

A total of 60 one-day-old male Cobb 500 broilers were obtained from the Integrated Management and Hatching Laboratory (Desert Road Facility, Alexandria governorate). A completely randomized design was used to randomly allocate the chickens into one of two groups: the control group (*n* = 30) and the SB-supplemented group (*n* = 30). Each group was represented by two replicates with 15 chicks per replicate, and each group of *n* = 15 was housed in separate, controlled environment pens in accordance with Directive 2010/63/EU, separated by wooden chipboard. Control chickens received water free from sodium butyrate, and SB supplemented received water containing 0.98 mg sodium butyrate (West Bengal Chemical Industries Ltd., India) per 1 ml of drinking water from days 1–28 [13]. We used male chickens only because, as recorded by [14], male chickens showed better performance in terms of more production.

The chicks were housed on litter on the floor at a depth of 5 cm and brooded at 32 °C for the first week, which was reduced by 3 °C weekly, then maintained at 20 °C by week four. Relative humidity was maintained at between 65% and 75%. Following standard healthcare regimes, the chicks were vaccinated against Newcastle disease and infectious bronchitis in their drinking water on the 8th and 18th days (using the Hitchner, IB, and HIPRAVIAR Clon live vaccine CL/79 clon). They were also vaccinated against infectious bursal disease (IBD) on the 14th day using an intermediate strain in drinking water [15]. Both groups received minerals and vitamins in their ad libitum feed (whole ingredients shown in Table 1) as per Nutrient Requirements of Poultry guidelines [16], with a starter diet provided on days 0 to 14 and a grower diet from day 15 until the end (Table 2). Fosfomycin antibiotics were added to their water as standard animal care (therefore, bacterial load was not investigated in the present study). The chicks had daily health checks and underwent cervical dislocation and decapitation on day 30.

**Table 1:** Experiment protocol

Age (days)	Treatment	Control
	**Drugs**	
1-2-3-4	Sodium butyrate 1 g/l 24 h	
5-6-7	Minerals 2 cm/l of drinking water	
	Vitamins 05 − 1 ml/l of drinking water	
	Sodium butyrate 1 g/l 24 h	
8-9-10-11-12-13	Sodium butyrate 1 g/l 24 h	
14–15	Sodium butyrate 1 g/l 24 h	
	Fosfomycin 1/2 g/l	
16–30	Sodium butyrate 1 g/l 24 h	
	**Vaccines**	
6	h120-B1hitchener (Izovac)	
14	Intermediate IBD	

Vitamins composition per liter: Vitamin A 100 000 000 I.U., Vitamin D3 20 000 000 I.U., and Vitamin E 20 000 mg (LOVIT AD3E LIQUID, Lohman Animal nutrition company, Germany)

Minerals composition per liter: Calcium 22,000 mg., Magnesium 10,000 mg., Sodium 7500 mg., Manganese 4800 mg, and Zinc 4000 mg (LOVIT PHOS LIQUID, Kaesler Animal nutrition company, Germany)

**Table 2:** Ingredients and nutrient composition (% dry mass) in broiler starter and grower food

Ingredient	Starter (%) days 1–14	Grower (%) days 15–30
Yellow corn	53.5	66.5
Soybean meal (48% starter mix)	34.38	24
Corn gluten	5.82	3.6
Salt	0.3	0.3
Dicalcium phosphate^A^	2.7	2.48
Vitamin premix^B^	0.3	0.3
Corn oil	3.00	2.82
Total	100%	100%
**Calculated values**		
Metabolizable Energy (ME) (kcal/kg)	2976.83	3096.31
Crude protein	22.97	18.07
Calcium	1.08	0.9
Available phosphorus	0.52	0.45
Methionine^C^	0.52	0.51
Lysine^D^	1.29	1.13

(A) Dicalcium phosphate, 18% granular phosphate, and 23% calcium. (B) Supplied per kg of diet: vitamin A 12,000 IU, vitamin D3 3,000 IU, vitamin E 40 mg, vitamin K3 3 mg, vitamin B1 2 mg, vitamin B2 6 mg, vitamin B6 5 mg, vitamin B12 0.02 mg, niacin 45 mg, biotin 0.075 mg, folic acid 2 mg, pantothenic acid 12 mg, manganese 100 mg, zinc 600 mg, iron 30 mg, copper 10 mg, iodine 1 mg, selenium 0.2 mg, cobalt 0.1 mg. (C) DL-Methionine, Met AMINO^®^ (DL-2- - amino-Y-methyl-oily acid)αamino-4-(methyl-thio)-butane acid, DL-methionine, by Feed Grade 99% (EU). (D) L-Lysine HCL 99% (Feed Grade) L-Lysine: 78.0% Min (Indonesia)

### Scanning electron microscopy

Samples from three small intestine regions were fixed in a mixture of 4% paraformaldehyde and 2.5 glutaraldehyde in PBS. All samples were kept at 4 °C for 48 h. After fixation, tissues were washed in 0.1 M Na-cacodylate buffer containing 5% sucrose and fixed with 1% osmic acid in 0.1 M Na-cacodylate buffer for 2 h. at room temperature [17, 18]. Then, they were washed with distilled water and dehydrated in ascending ethanol grades series for 15 min per ethanol concentration. The samples were then dried at a critical point with carbon dioxide (JFD-300; JEOL, Tokyo, Japan) [19], placed under copper stubs with a double face carbon tape, and coated with gold-palladium (80/ 20) to a thickness of 400 Å in a sputter-coating unit (JFC-1100 E) [20–22]. Tissues were examined from various angles and imaged with a JEOL SEM (JSM-IT200) operating at 15 kV at the Faculty of Science, Alexandria University (Egypt).

### RNA extraction and quantitative real-time PCR (qRT-PCR) analysis of host defense peptides

Total RNA was extracted from the intestinal mucosae from the three parts of the intestine in both groups using Sepasol-RNA I Super (Nacalai Tesque Co. Inc. Japan), according to the manufacturer’s instructions. The RNA samples were treated with RQ1 RNase-free DNase (Promega Co., Madison, WI, USA) on a programmable thermal controller (PTC-100; MJ Research, Waltham, MA, USA) at 37 °C for 45 min and then at 65 °C for 10 min and the concentration and purity were measured using a spectrophotometer. The RNA samples were reverse transcribed using ReverTra Ace (Toyobo Co. Ltd., Osaka, Japan) according to the manufacturer’s instructions. Reverse transcription (RT) was performed at 42 °C for 30 min, followed by heat inactivation at 99 °C for 5 min using the programmable thermal controller (PTC-100; MJ Research, USA). The expression of AvBDs was examined by qRT-PCR using DETECTING DT lite 4 THERMOCYCLER (DNA TECHNOLOGY, Research, and Production, Moscow, Protvino, Russia) following the MIQE guidelines [23]. Target gene expression was examined by qRT-PCR using chicken-specific primers (Table 3). The PCR mixture (10 µL) consisted of 0.5 µL cDNA, 1 x Thunderbird SYBR qPCR Mix (Toyobo Co. Ltd. Japan), and 250 nM of each primer. Target genes were amplified under the following conditions: heating at 95 °C for 120 s, followed by 40 cycles of 95 °C for 10 s with annealing temperatures, and extension at 72 °C [24, 25]. The qRT-PCR data were analyzed using the 2-DDCT method to calculate the relative expression in each sample [26]. All qRT- PCR products were confirmed by electrophoresis on 2% (w/v) agarose gel with 0.6% ethidium bromide and observed on a Transilluminator (NTM-10E; UVP LLC, Upland, CA, USA) [25].

**Table 3:** Primers were used in this study to detect the different genes using real-time PCR

Gene		Primer sequence (5ʹ − 3ʹ)	Accession # (reference)	*N*
*AvBD1*	F: GATCCTCCCAGGCTCTAGGAAG	R: GCCCCATATTCTTTTGC	NM_204993(a)	55
*AvBD2*	F: GTTCTGTAAAGGAGGGTCCTGCCAC	R: ACTCTACAACACAAAACATATTGC	NM_001201399(a)	55
*AvBD10*	F: TGGGGCACGCAGTCCACAAC	R: CATGCCCCAGCACGGCAGAA	NM_001001609(a)	58
*AvBD12*	F: GGAACCTTTGTTTCGTGTTCA	R: GAGAATGACGGGTTCAAAGC	NM_001001607(a)	55
*RPS17*	F: AAGCTGCAGGAGGAGGAGAGG	R: GGTTGGACAGGCTGCCGAAGT	NM_204217 (a)	62

### Morphometric examination

The morphometric examination of the SEM showed figures of small intestine parts: the duodenum, jejunum, and ileum of each bird. That was analyzed using ImageJ software (NIH) [17, 27]. The measurements of the villus height, villus, and width were calculated.

### Statistical analysis

The statistical analysis was made using a t-test to compare the control and treated groups in terms of their effects on different variables under study. According to SAS 2004, an analysis of variance was made to compare the different studied variables in the same group [28, 29].

## Results

### Chicks’ bodyweight

The body weight was recorded in (Table 4). The frame weight of the bird varied significantly (*P* < 0.01) between the control and treatment groups of the studied chicken. In particular, in the 4th and 5th weeks of the experiment, the mean weight of the treatment group reached 1637 gm, while in the control group, it was 1530 gm.

**Table 4:** Bodyweight (gm) of Cobb’s broilers at the different periods of the experiment

Age (Week)	Treatment	Control	t-test
1st Week	85 ± 5.82	80.40 ± 4.83	1.45^NS^
2nd Week	240.9 ± 9.45	239.2 ± 3.44	1.22^NS^
3rd Week	359.15 ± 10.15	341.2 ± 8.15	3.45^*^
4th Week	1560 ± 15.45	1430 ± 15.29	6.55^**^
5th Week	1637 ± 16.44	1530 ± 15.30	6.74^**^

NS = Non-significant at (*P* > 0.05). * = Significant at (*P* < 0.01). ** = Significant at (*P* < 0.01)

### Small intestine parameters

The weight and length of the small intestine differed significantly (*P* < 0.05) among the control and treatment groups. In the control group, the small intestine weight and length were 36.28 gm and 127.57 cm; the effects in the treatment group showed that the weight and length of the small intestine increased to 75.14 gm and 151.42 cm (Table 5).

**Table 5:** Small intestine parameters of the 30-day Cobb’s broilers of the control and treatment groups

	Control	Treatment	t-test
Weight (gm)	36.28 ± 3.60^b^	75.14 ± 4.15^a^	7.55^**^
Length (cm)	127.57 ± 12.27 ^b^	151.42 ± 5.24^a^	8.44^**^
Width (cm)	1.75 ± 0.25^a^	1.75 ± 0.25^a^	1.55^NS^

NS = Non-significant at (*P* > 0.05). * = Significant at (*P* < 0.01). ** = Significant at (*P* < 0.01)

### Scanning electron microscopy

**Fig. 1:**
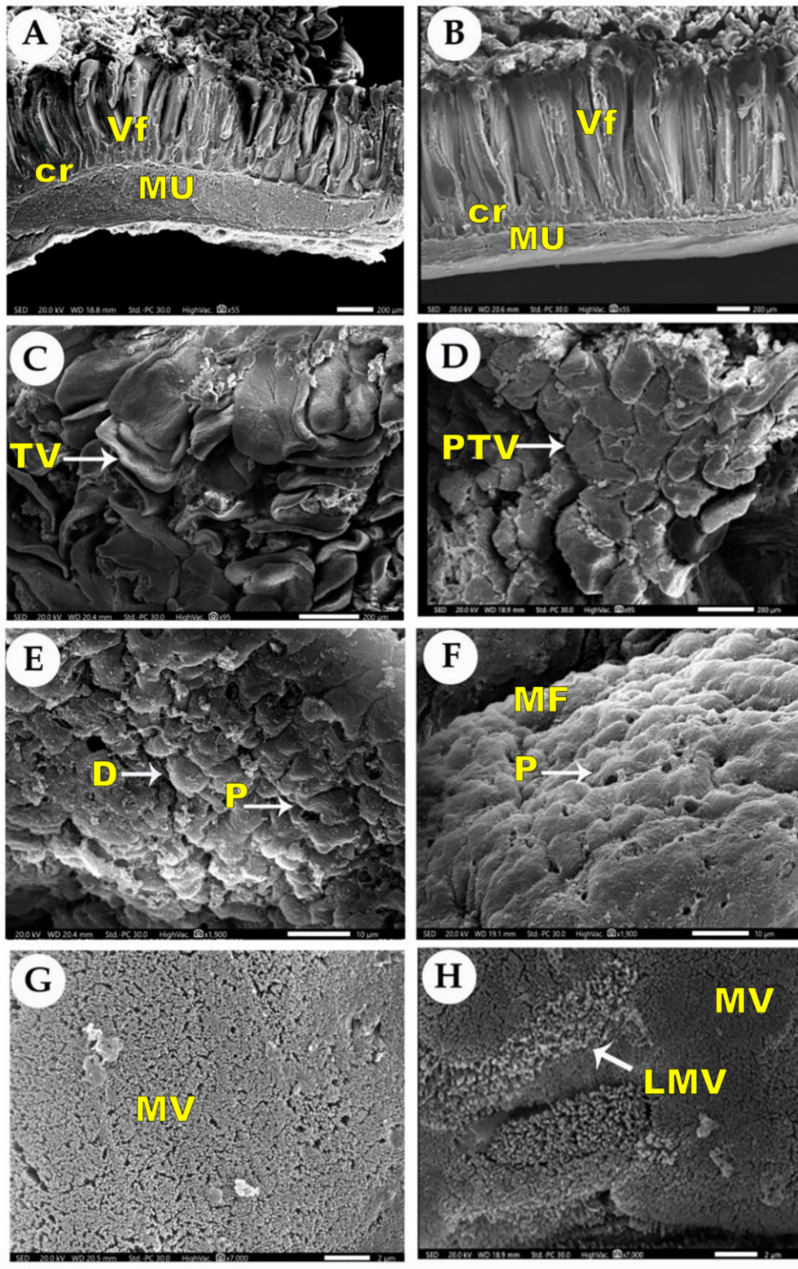
Scanning electron micrograph of the duodenum villi of the 30 days Cobb’s broilers (Views **A**, **C**, **E**, and **G** from the control group - Views **B**, **D**, **F**, and **H** from the treatment group), (Views **A**-**B**) lateral view of the duodenal villi, (Views **C**-**D**) dorsal view of the tips of the duodenal villi, (Views **E**-**F**) magnification of tip of the duodenal villi, and (Views **G**-**H**) magnification of the microvilli. The tongue villi with a recess between them (Vf), intestinal crypt (Cr), muscular layer (Mu), thin, slightly curved tips of duodenal villi (TV), the rectangular or polygonal outline of the tips of the duodenal villi (PTV), dome shapes of the epithelium lining (D), microfolds of the epithelium lining (MF), pores of the goblet cells (P), microvilli (MV), and long microvilli (LMV)

**Fig. 2:**
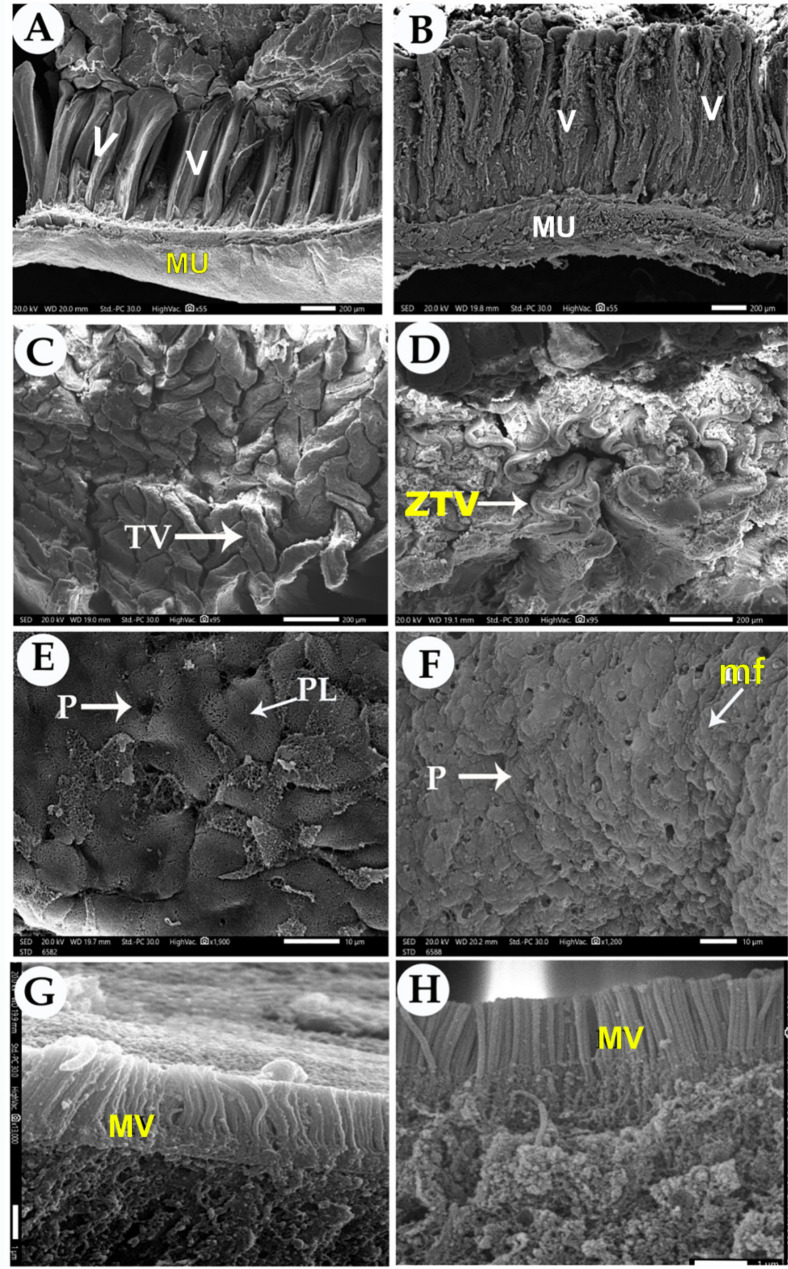
Scanning electron micrograph of the jejunum villi of the 30-day Cobb’s broilers (Views **A**, **C**, **E**, and **G** from the control group - Views **B**, **D**, **F**, and H from the treatment group). (Views **A**-**B**) lateral view of the jejunal villi, (Views **C**-**D**) dorsal view of the tips of the jejunum villi, (Views **E**-**F**) magnification of the tip of the jejunal villi, and (Views **G**-**H**) magnification of the microvilli. The tongue villi (VT), muscular layer (Mu), thin rectangular outline of the jejunal villi tips arranged at a wave pattern (TV), the zigzag form of the jejunal villus (ZTV), the epithelium lining was polygonal shape (PL), microfolds of the epithelium lining (MF), and the pores of the goblet cells (P), microvilli (MV)

**Fig. 3:**
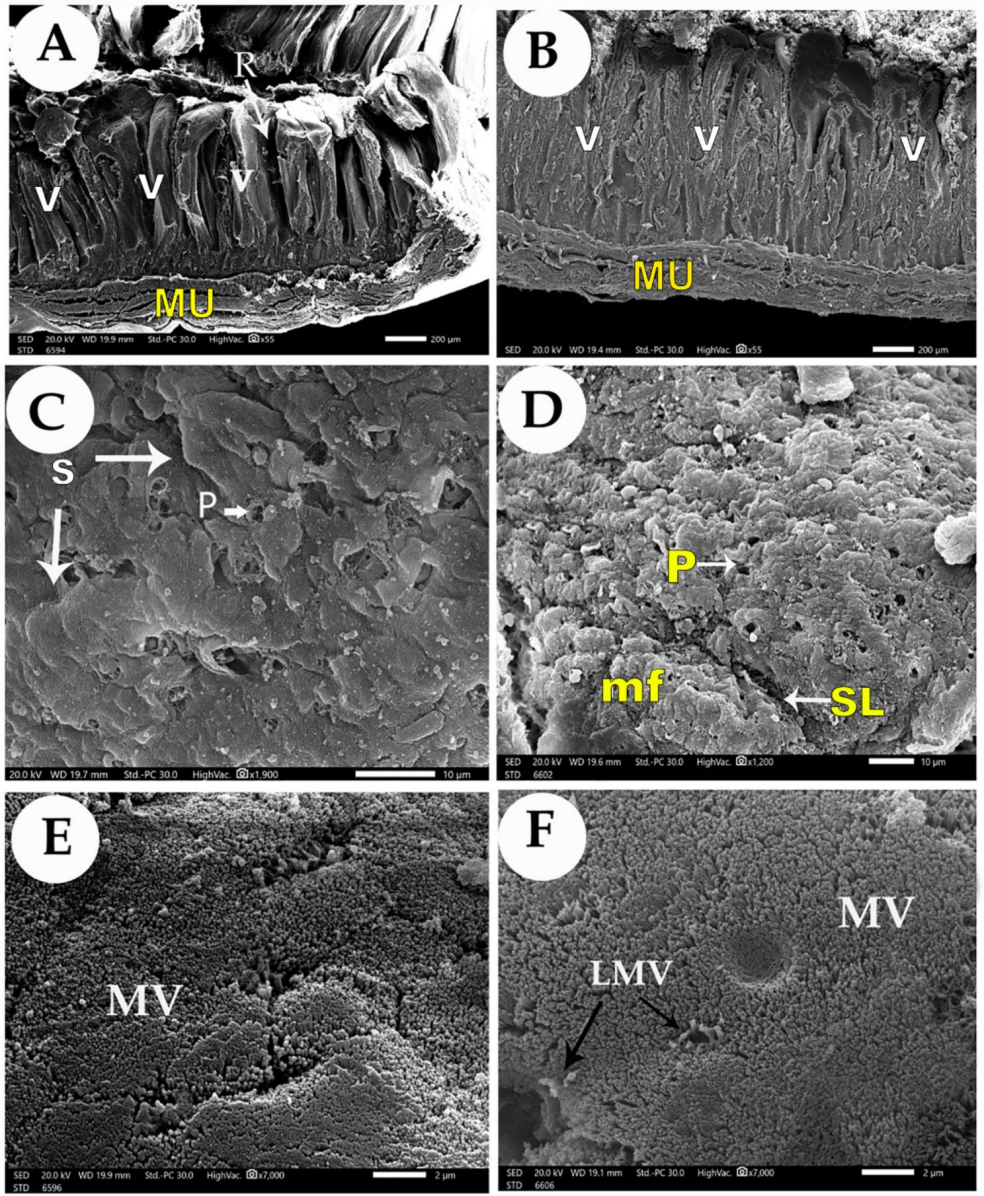
Scanning electron micrograph of the ileum villi of 30-days Cobb’s broilers (Views **A**, **C**, and **E** from the control group - Views **B**, **D**, and **F** from the treatment group). (Views **A**-**B**) lateral view of the ileum villi, (Views **C**-**D**) dorsal view of the tips of the jejunum villi, and (Views **E**-**F**) and magnification of the microvilli. The tongue villi (VT) with recesses between them (R), the muscular layer (Mu), the epithelium lining were scale-like shape (PL), the microfolds of the epithelium lining (MF) and separated by sulci (S), the pores of the goblet cells (P), the microvilli (MV), long microvilli (LMV)

In the control group, the intestinal villi were tongue-shaped in the three parts of the small intestine; they aligned with each other in the jejunum, and recesses were present between them in the duodenum and ileum. In the treatment group, the intestinal villi appeared long and tongue-shaped and aligned with each other in the jejunum and ileum, while the duodenum had long finger-like villi and was aligned with each other (Figs. 1, 2 and 3).

The tips of the villi in the control group were thin and slightly curved, with a prominent central part at the duodenal villi; they were thin, rectangular outlines in the jejunal and ileal villi and were arranged in a wave pattern at the jejunal villi. The tips of the villi in the treatment group had thick rectangular or polygonal outlines in the duodenal and ileal villi, and they appeared as a zigzag form in the jejunal villi (Figs. 1, 2 and 3).

In the control group, the epithelium lining contained a few large pores of goblet cells in the three parts of the small intestine. However, the lining of the duodenal villi showed dome shapes, the jejunal villi epithelium lining was polygonal, and the ileal villi appeared scale-like. In the treatment group, the epithelium lining had irregular microfolds that carried small dome shapes in the duodenal and ileal villi and carried sulci at the ileal villi. In the jejunal villi, the irregular microfolds appeared as stratified bands. The pores of goblet cells were large in number and had different diameters in the three parts of the small intestine. The duodenal microvilli were short in the control group, while they were islands of long microvilli in the treatment group. The jejunal villi appeared more aligned in the treatment group than in the control group, and the ileal villi were short in the control group. While they were aligned with each other, solitary long microvilli in the treatment group appeared (Figs. 1, 2 and 3).

The morphometric analysis of SEM showed that the treatment group with sodium butyrate showed a high difference (*P* < 0.01) in the small intestine villi height and width compared to the control group. The rate of increase in the length of intestinal villi in the treatment group compared to the control group was 48.29%, 52.9%, and 86.6% in the duodenum, jejunum, and ileum. The high value of intestinal villi height was recorded at the duodenum (989.6 μm), while the low value was at the ileum (889.32 μm). The rate of increase of the intestinal villi width in the treatment group compared to the control group was 68.86%, 55%, and 66% in the duodenum, jejunum, and ileum. The high value of intestinal villi width was recorded in the duodenum (120.2 μm), while the low value was at the jejunum (104.2 μm) (Table 6).

**Table 6:** Scanning electron microscopic measurements of the villi height and villi width of small intestine at the control and treated groups

Groups	Part of the small intestine	VH	VW
		Mean &Std. Error	Mean& Std. Error
Control	Duodenum	664.52 ± 25.38 ^d^	71.22 ± 3.03 ^d^
	Jejunum	638.15 ± 13.46 ^f^	67.24 ± 1.96 ^d^
	Ileum	469.03 ± 12.19 ^e^	64.2 ± 4.45 ^d^
Treatment	Duodenum	989.6 ± 29.12 ^b^	120.24 ± 6.84 ^a^
	Jejunum	976.83 ± 14.96 ^a^	104.20 ± 8.94 ^c^
	Ileum	889.32 ± 32.05 ^c^	106.95 ± 4.23 ^b^
Rate of increase of the measurements of the treatment group from the control group			
	Duodenum	325.1 μm (48.9%)	49 μm (68.8%)
	Jejunum	338.1 μm (52.9%)	37 μm (55%)
	Ileum	420 μm (86.6%)	42.4 μm (66%)

Means within the same column carrying different superscript are significantly different at *p* < 0.05

### Real-time PCR results of the different genes among the three parts of the small intestine

**Fig. 4:**
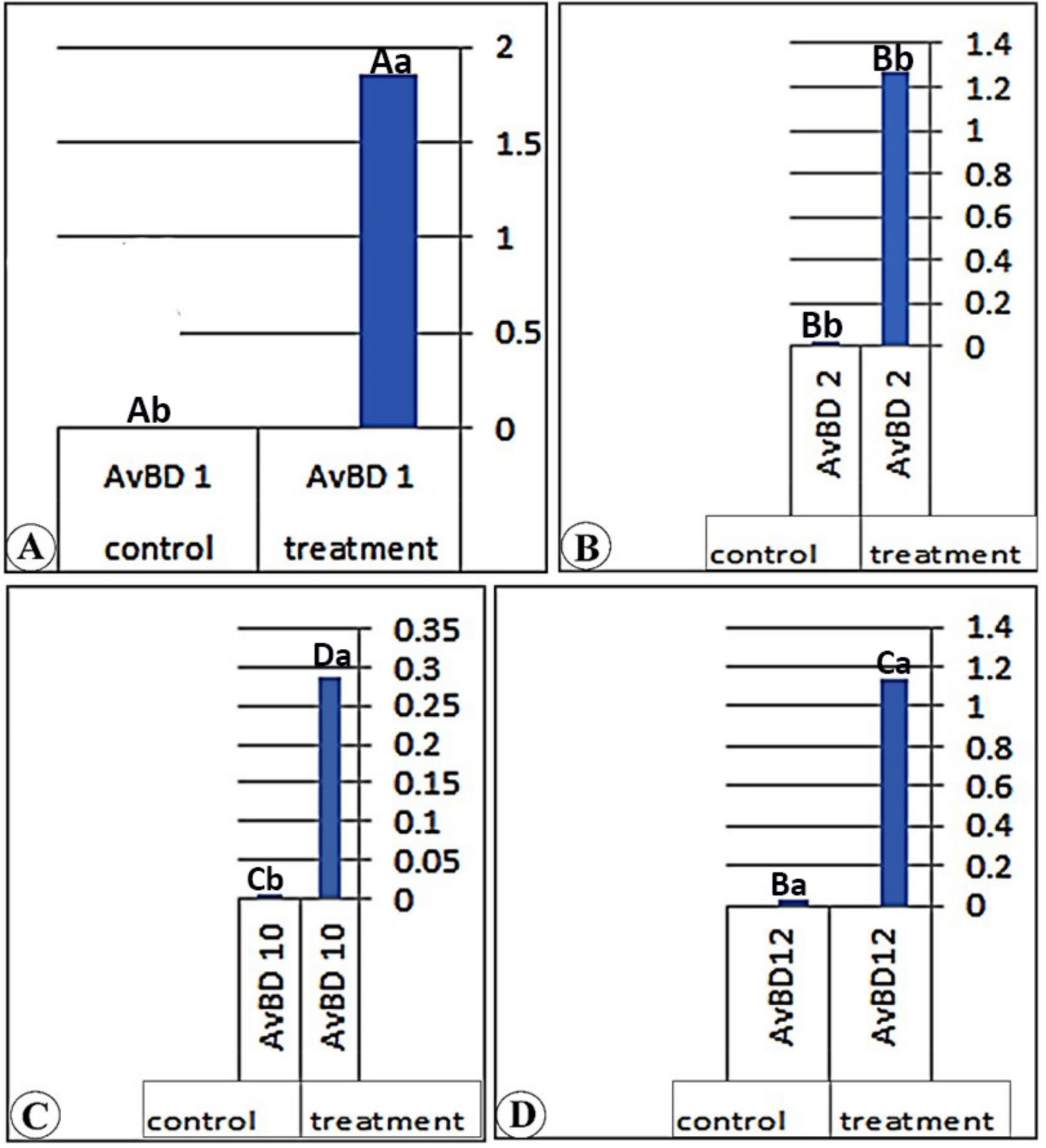
A chart (Views A-D) demonstrates the real-time PCR results of (AvBD 1, 2, 10, and 12) gene expression of the duodenum in the treatment and control group

**Fig. 5:**
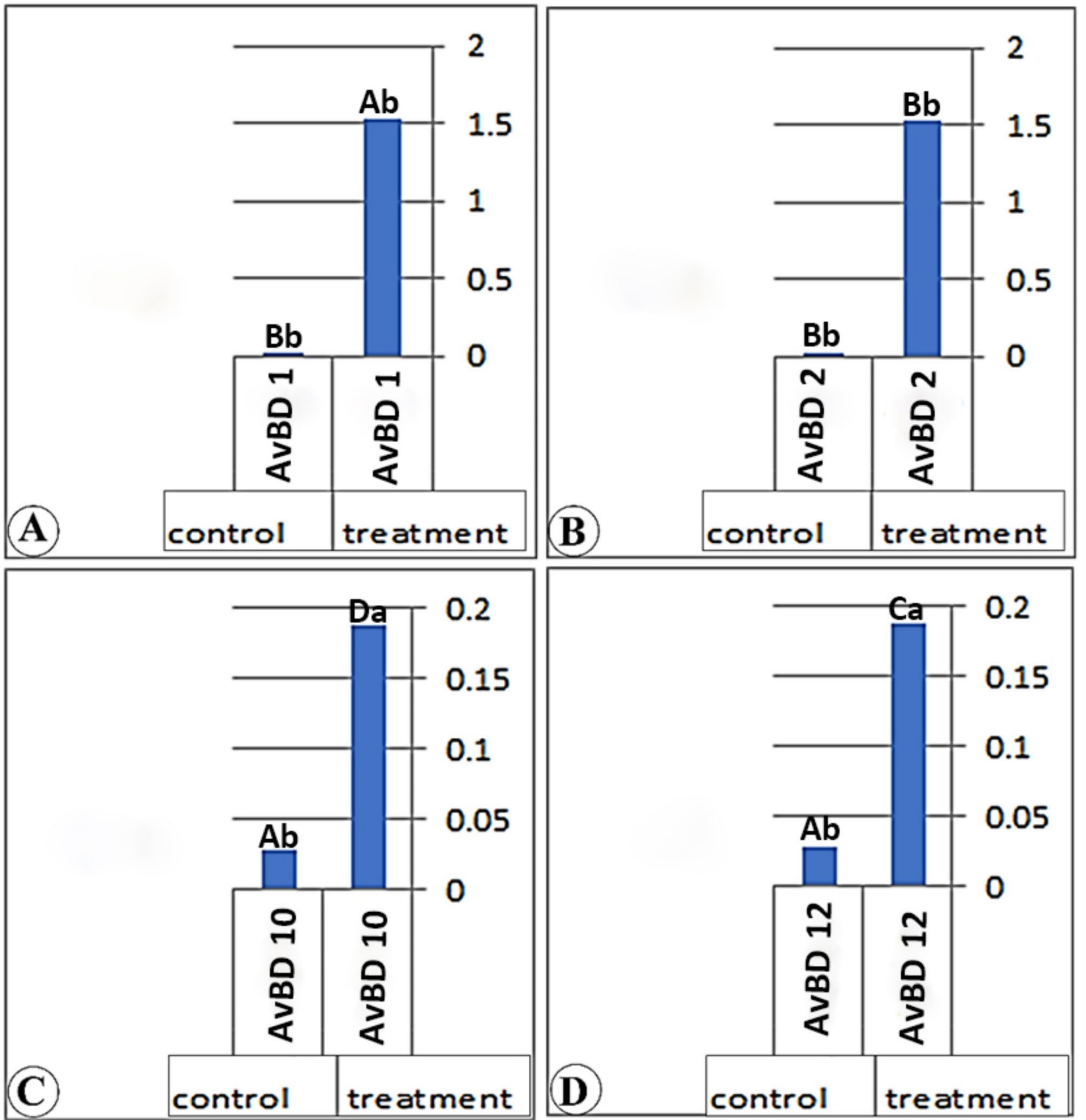
A chart (Views **A**-**D**) demonstrates the real-time PCR results of (AvBD 1, 2, 10, and 12) gene expression of the jejunum in the treatment and control group

**Fig. 6:**
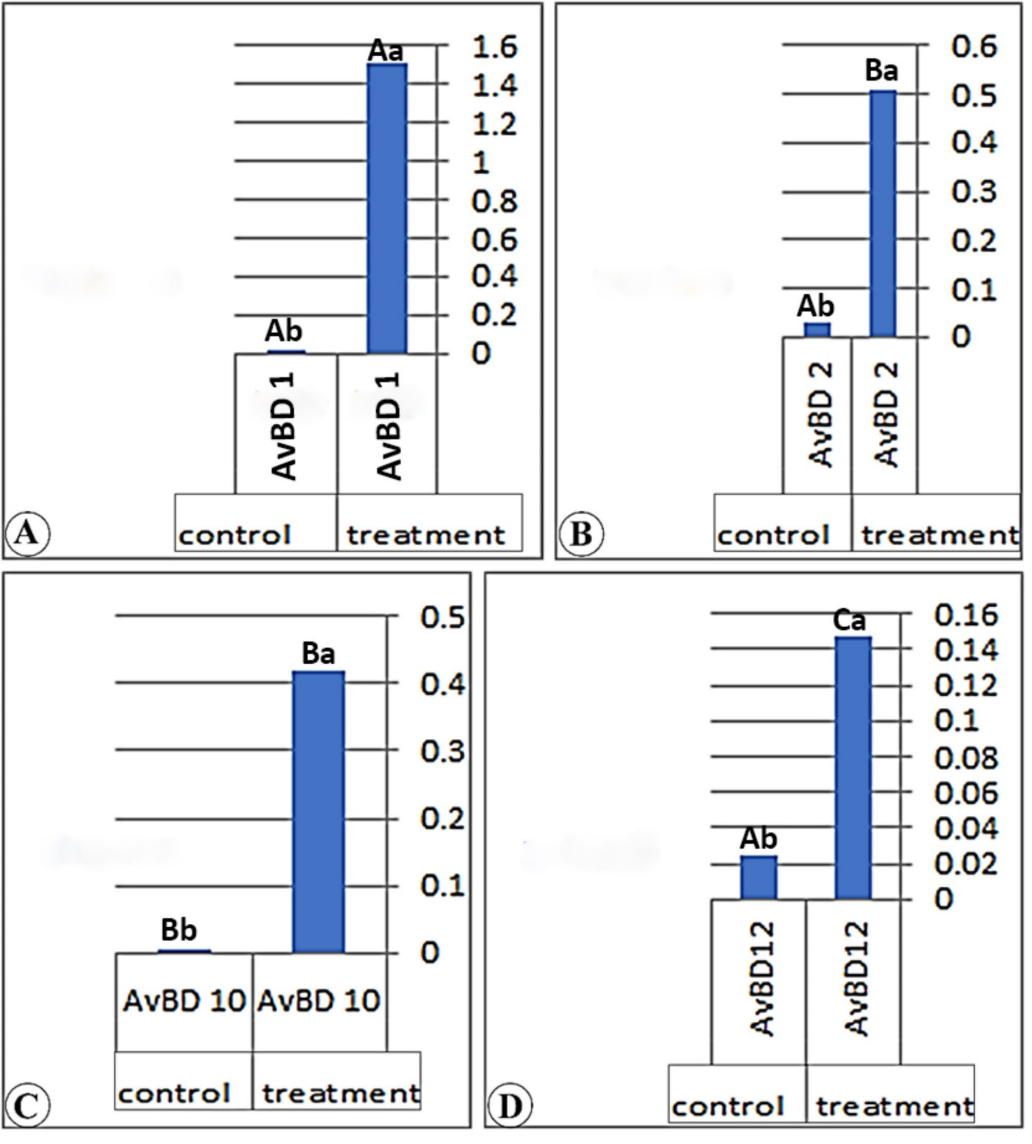
A chart (Views **A**-**D**) demonstrates the real-time PCR results of (AvBD 1, 2, 10, and 12) gene expression of the ileum in the treatment and control group

Our consequences have determined in (Table 7) and (Figs. 4, 5 and 6) that the real-time PCR of the various genes (AvBD 1), (AvBD 2), (AvBD 12), and (AvBD 10) fluctuate significantly (*P* < 0.01). The expression of the various genes was higher in the treatment group than in the control group. The better expression of (AvBD 1), (AvBD 2), and (AvBD 12) has been determined in the duodenum of the treatment group. The better expression of (AvBD 10) was determined in the jejunum of the treatment group.

**Table 7:** Real-time PCR results from different genes among different anatomical parts of the Cobb’s broilers intestine

Gene	Duodenum		Jejunum		Ileum	
	Treatment	Control	Treatment	Control	Treatment	Control
AvBD 1	1.85 ± 0.02 ^Aa^	0.01 ± 0.001^Ab^	1.53 ± 0.01^Ab^	0.01 ± 0.01^Bb^	1.51 ± 0.01^Aa^	0.01 ± 0.01^Ab^
AvBD 2	1.27 ± 0.01^Bb^	0.02 ± 0.001^Bb^	0.34 ± 0.01^Bb^	0.02 ± 0.01^Bb^	0.51 ± 0.01^Ba^	0.03 ± 0.01^Ab^
AvBD 10	0.28 ± 0.01^Da^	0.003 ± 0.000^Cb^	0.43 ± 0.01^Da^	0.03 ± 0.01^Ab^	0.42 ± 0.01^Ba^	0.00 ± 0.00^Bb^
AvBD12	1.14 ± 0.01 ^Ca^	0.02 ± 0.001^Bb^	0.19 ± 0.01^Ca^	0.03 ± 0.01^Ab^	0.15 ± 0.01^Ca^	0.02 ± 0.01^Ab^

Capital litters: Indicated that: Means within the same column of different litters are significantly different at (*P* < 0.5). Small litters: Indicated that: Means within the same rows of different litters are significantly different at (*P* < 0.05)

In the duodenum of the treated group, the gene expressions are higher than those in the control group by 205.32, 58, 51.44, and 872.6 folds for (AvBD 1, 2, 10, and 12) genes, respectively. In the jejunum, the gene expression is higher than in the control group by 291.12, 13.7, 6.7, and 16.5 folds for (AvBD 1, 2, 10, and 12) genes, respectively. In the ileum, the gene expression is higher than that in the control group by 282.6, 15.6, 5.9, and 710.7 folds for (AvBD 1, 2, 10, and 12) genes, respectively. The gene expression of the (AvBD 1) gene is higher in the jejunum of the treated chicken than in other small intestinal parts. The gene expression of (AvBD 2, 10, and 12) is higher in the duodenum of the treated chicken than in other small intestinal parts.

## Discussion

The Cobb 500 male showed an ability to respond to increasing amino acid density as the bird aged and any beneficial effects of feeding high-amino acid diets early in life Corzo 2010. Butyric acid has a disagreeable smell and potentially unstable volatility; sodium butyrate has generally been used in broiler production. In the gastrointestinal system of chickens, sodium butyrate is easily converted into an effective component. The development of the intestinal mucosa and morphological structures are considered to be enhanced by sodium butyrate supplementation, and the growth of the symbiotic intestinal microbiota is thought to be moderated. As a result, dietary SB may be beneficial for the physiological function and health of the intestines [7].

Our scanning electron microscopy results revealed that the shape, tip, and microvilli of the villi changed in the treated group with SB, which increased their surface area. The zigzag microfolds of the epithelium and the two types of microvilli modulation enhanced the nutrient absorption more than the villi arranged in parallel. The zigzag flux in the small intestine permits nutrients to take a long passage through the alimentary tract compared to the straight route [30]. Longer villi were thought to sustain larger surface areas, enabling higher absorption capacities and healthier intestinal development, resulting in the gut’s optimal state [31]. Our study reported that the morphometrics of the villus height and crypt depth were improved with sodium butyrate supplementation to the broiler, similar to those described by [32, 33]. The higher villus might increase the surface area of the absorption in the luminal capillaries and subsequently produce sufficient digestive enzymes and nutrients transported on the surface of the villi [33, 34]. The intestinal villi established a plate-like shape in the duodenum, a wave-like shape in the jejunum, and a tongue-like shape in the ileum at 30 days of age via the common plate-like villi at 10 days of age [35]. Two types of obliquely elongated plate-like villi showed a zigzag arrangement, connecting at an angle of 40° to 60° like an oblique T-shape. This villous arrangement would be more effective for nutrient absorption by inducing a long, zigzag flow of ingesta. [32] mentioned the importance of sodium butyrate in improving the intestinal development, morphological structure, and biological functions of broilers through modulation of the microbial community, which seems to be optimized for gut health at higher doses (800 mg/kg) of sodium butyrate. The sodium butyrate enhanced the intestinal structure by stimulating (*P* < 0.05) increased (Pdiets < 0.10) ileal villus height. In addition, more irregular leaf-shaped villi and mucus secretion and significantly fewer erosions were demonstrated by scanning electron microscopy.

Sodium butyrate has a direct bactericidal effect due to it lowering the pH of the crop, gizzard, and upper part of the intestine [36]. After ingestion, sodium butyrate converts into butyric acid and is absorbed by enterocytes. It hastens the growth of enterocytes and villus elongation, which increases the villi height and crypt depth [37, 38].

Sodium butyrate converts into butyric acid after ingestion and is absorbed by enterocytes. It hastens the growth of enterocytes and villus elongation, which increases the villi height and crypt depth [37, 38]. Butyrate is an inducer of hen HDPs in number one monocytes, bone marrow duodenum, jejuna, and ileum [39]. Butyrate has the capability for similar improvement as a handy antibiotic-opportunity approach to enhance host innate immunity and disorder resistance [12].

In our work, we noticed that the expression of various genes (AvBD 1), (AvBD 2), (AvBD 12), and (AvBD 10) was higher in the treatment group than in the control group. The better expressions (AvBD 1), (AvBD 2), and (AvBD 12) were determined in the duodenum of the treatment group, while the better gene expression (AvBD 10) was in the jejunum of the treatment group. While [40, 41] recorded that (AvBD10) from β-defensins was the only gene slightly induced by sodium butyrate.

## Conclusion

Sodium butyrate enhanced the morphological characteristics of the small intestine of the broiler chicks, which are reflected in the intestinal villi characteristics of length and width. And micro-anatomical structures that accelerate the absorption process. The better expression (AvBD 1), (AvBD 2), and (AvBD 12) was determined in the duodenum of the treatment group, while the better gene expression (AvBD 10) was in the jejunum of the treatment group. Sodium butyrate enhanced the chicks’ growth and small intestine parameters, modified the morphology of the intestinal villi, and improved the humoral part of innate immunity, which may help as SB is used in the routine feed program in our country. The study’s limitations included using only one concentration of sodium butyrate and four genes. In the future, we plan to focus on more than four genes and combine sodium butyrate plant extract with turmeric or thyme to see if it affects bird immunity.

## Data Availability

The datasets used and analyzed during the current study are available from the corresponding author upon reasonable request.
